# In Silico Analysis Reveals the Modulation of Ion Transmembrane Transporters in the Cerebellum of Alzheimer’s Disease Patients

**DOI:** 10.3390/ijms241813924

**Published:** 2023-09-10

**Authors:** Simone D’Angiolini, Maria Sofia Basile, Emanuela Mazzon, Agnese Gugliandolo

**Affiliations:** IRCCS Centro Neurolesi “Bonino-Pulejo”, Via Provinciale Palermo, Contrada Casazza, 98124 Messina, Italy; simone.dangiolini@irccsme.it (S.D.); mariasofia.basile@irccsme.it (M.S.B.); agnese.gugliandolo@irccsme.it (A.G.)

**Keywords:** Alzheimer’s disease, cerebellum, in silico analysis, ion transmembrane transporters

## Abstract

Alzheimer’s disease (AD) is the most common neurodegenerative disorder. AD hallmarks are extracellular amyloid β (Aβ) plaques and intracellular neurofibrillary tangles in the brain. It is interesting to notice that Aβ plaques appear in the cerebellum only in late stages of the disease, and then it was hypothesized that it can be resistant to specific neurodegenerative mechanisms. However, the role of cerebellum in AD pathogenesis is not clear yet. In this study, we performed an in silico analysis to evaluate the transcriptional profile of cerebellum in AD patients and non-AD subjects in order to deepen the knowledge on its role in AD. The analysis evidenced that only the molecular function (MF) “active ion transmembrane transporter activity” was overrepresented. Regarding the 21 differentially expressed genes included in this MF, some of them may be involved in the ion dyshomeostasis reported in AD, while others assumed, in the cerebellum, an opposite regulation compared to those reported in other brain regions in AD patients. They might be associated to a protective phenotype, that may explain the initial resistance of cerebellum to neurodegeneration in AD. Of note, this MF was not overrepresented in prefrontal cortex and visual cortex indicating that it is a peculiarity of the cerebellum.

## 1. Introduction

Alzheimer’s disease (AD) represents the most common neurodegenerative disorder and the most common cause of dementia [1]. The most important AD clinical symptoms are memory impairments and cognitive deficits [2]. However, non-cognitive impairments, including motor dysfunction, are also associated with AD [2]. The majority of AD patients develop clinical symptoms after 65 years of age (late-onset AD), whereas only about 5–10% of patients show an earlier onset (early-onset AD) [1,3,4]. Most early-onset AD patients do not exhibit a clear autosomal inheritance pattern, although there are rare autosomal dominant forms of AD [4]. Over 300 pathogenic mutations in the genes presenilin 1 (*PSEN1*), presenilin 2 (*PSEN2*), and amyloid precursor protein (*APP*) have been identified in autosomal dominant AD cases, further establishing the crucial role of amyloid in AD [3,5,6,7]. However, the genetic predisposition of the non-Mendelian form of AD is significant also for late-onset AD patients [4]. Indeed, the heritability of non-Mendelian, late-onset AD is estimated to be nearly 60–80%, with the apolipoprotein E (*APOE*) ε4 allele as the most frequent risk factor [8,9]. Although different additional genetic risk loci for late-onset AD have been identified via genome-wide association studies (GWAS), it seems that a significant part of the genetic variance beyond the *APOE* risk continues to be hidden [10].

Currently, diagnostic methods for AD primarily rely on neurocognitive tests, brain imaging techniques, and cerebrospinal fluid assays [11].

At present, the exact mechanism underlying AD is still unknown, and there is no yet effective treatment able to reverse or delay AD progression [7,12,13]. Among the US Food and Drug Administration (FDA)-approved drugs for the treatment of AD there are the cholinesterase inhibitors donepezil, rivastigmine, and galantamine; the uncompetitive N-methyl-D-aspartate (NMDA) receptor modulator memantine; a combination of memantine and donepezil; and the monoclonal antibodies targeting β-amyloid (Aβ) aducanumab and lecanemab [12,14,15,16,17,18]. 

AD is characterized by progressive neuronal and synaptic loss and by the presence of extracellular Aβ plaques and intracellular neurofibrillary tangles consisting of hyperphosphorylated tau in the brain [19,20,21,22,23]. Synaptic loss and neuronal degeneration cause memory impairment, cognitive decline, and behavioral dysfunctions in AD patients [15]. Amyloidosis decreases acetylcholine synthesis and release, and NMDA receptors hyperactivation, with the consequent rise in intra-neuronal calcium (Ca^2+^) levels resulting in excitotoxic neuronal death [15]. Amyloid plaques are initially present in the cerebral cortex, however, with disease progression they appear in subcortical regions and in the last stages in the cerebellum [24]. 

The innate immune system plays a main role in AD. In particular, microglia, belonging to the innate immune system, is deeply involved in the onset and progression of AD pathology and can interact with Aβ. Moreover, modulating the innate immune response represents a promising strategy for AD [25].

Considering that the cerebellum is regarded as relatively unaffected during the preclinical stage of AD, it is plausible to hypothesize that it may exhibit resistance to specific neurodegenerative mechanisms [26]. The role of the cerebellum in AD has been undervalued for a long time [24]. However, recent evidence suggests that, in addition to its key role in the coordination of voluntary motor activity and motor learning, it exerts also non-motor functions, such as the regulation of cognitive and behavioral processing, thus suggesting its potentially important role in AD [24,27,28]. Moreover, Aβ deposits were found in the cerebellum of AD patients [24] and it was shown to be a region vulnerable to Aβ toxic damage [28]. Therefore, it is essential to comprehend the development of AD pathology in the cerebellum, elucidate its role in AD pathology, and investigate how cerebellar changes impact cognition.

Interestingly, it was reported in AD brains an impairment of cell ion homeostasis, probably triggered by Aβ, that may affect the electrophysiological activity of brain cells, contributing to the AD pathophysiology [29]. It has been shown that Aβ accumulation results in Ca^2+^ dyshomeostasis mainly by rising intracellular Ca^2+^ concentrations, and also that intracellular Ca^2+^ levels can modulate both the APP processing and Aβ production and the formation of neurofibrillary tangles [30]. In addition, it has been suggested that an imbalance of sodium (Na^+^) can also be present in AD brains following the accumulation of Aβ [30]. 

The aim of this in silico study was to evaluate the transcriptomic profile of the cerebellum of AD patients compared to non-AD subjects in order to clarify its role in AD.

## 2. Results

**Figure 1:**
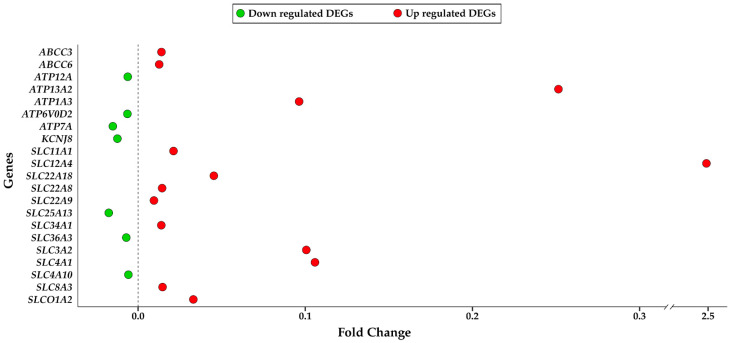
Dot plot presenting the Fold change of DEGs involved in the MF “active ion transmembrane transporter activity”. In the y-axis, the list of DEGs is reported and in the x-axis their levels of fold change are reported. Red dots are related to the genes more expressed in an AD condition; on the contrary, green dots are related to the genes more expressed in a healthy condition.

At first, we compared cerebellum expression data of non-AD and AD groups. We accepted, as differentially expressed genes (DEGs), all those genes with a q-value < 0.05. q-value for each gene was calculated using the false discovery rate method to adjust the *p*-value. The use of the q-value instead of the *p*-value lead us to reduce the number of false positives. Comparison of transcriptomic data related to cerebellum of healthy group (non-AD) and AD affected group resulted in 572 DEGs. Among these 572 genes, we performed gene ontology overrepresentation analysis (ORA) to identify any highlighted or significantly represented molecular functions (MF), biological processes (BP), and cellular components (CC). Through ORA we explored the ontologies in which the pathological condition had a bigger impact. As already reported for DEGs analysis, also for ORA, the threshold of the q-value to consider an ontology modified in a statistically significant way was set to 0.05. The analysis revealed that, among the MF, the only one that was overrepresented was the GO:0022853 related to “active ion transmembrane transporter activity”. The presence of only one enriched MF is an important result because it highlights how the transport of ion across the membrane is altered by the AD condition in the cerebellar area. We studied the 21 DEGs involved in this MF. The list with their associated fold changes is presented in Figure 1.

**Figure 2:**
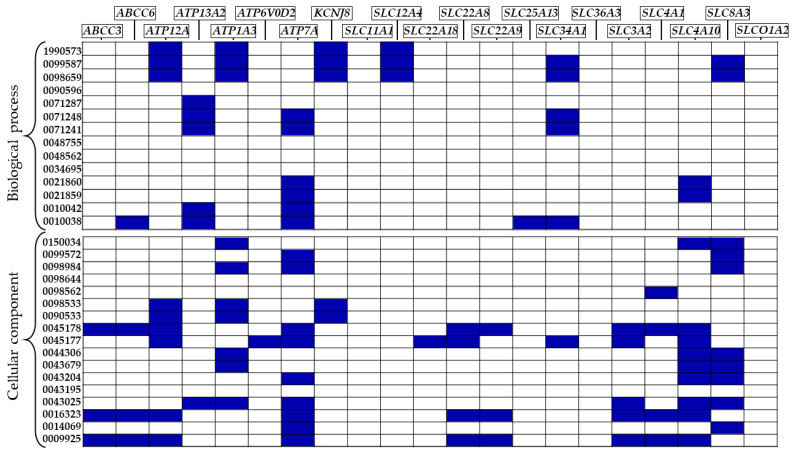
Plot showing the occurrence of the 21 DEGs involved in the MF “active ion transmembrane transporter activity” in the different biological processes and cellular components resulted overrepresented. On the left, the list of GO shows that the upper part is related to the biological processes and the lower part is related to the cellular components. In the graph, the list of the genes is reported and each part reported in blue in the plot indicates the presence of a specific gene in an enriched ontology.

Then, we evaluated if the 21 DEGs reported in Figure 1 were present in any overrepresented BP and CC to observe if the imbalance in ion transport activity was observable also through these ontologies. The number of BP and CC resulted overrepresented was 14 and 17, respectively. The 14 BP are, respectively, “GO:0021860”, “GO:0048755”, “GO:0021859”, “GO:0071287”, “GO:0010042”, “GO:0034695”, “GO:1990573”, “GO:0098659”, “GO:0099587”, “GO:0071248”, “GO:0071241”, “GO:0090596”, “GO:0048562”, and “GO:0010038”. The 17 CC overrepresented are “GO:0090533”, “GO:0098644”, “GO:0098533”, “GO:0043195”, “GO:0043679”, “GO:0044306”, “GO:0043204”, “GO:0016323”, “GO:0098562”, “GO:0009925”, “GO:0045178”, “GO:0150034”, “GO:0043025”, “GO:0098984”, “GO:0014069”, “GO:0099572”, and “GO:0045177”. Figure 2 reports on all the GO above and for each of them, it indicates which DEGs of the 21 showed in Figure 1 are included. 

In Figure 2, we can observe how the 21 DEGs of Figure 1 are also present in the majority of the BP and CC overrepresented. In this sense, the overrepresented ontologies are connected themselves by means of the 21 shared DEGs. Overrepresented BP and CC that include at least 1 of the 21 DEGs are reported in Table 1. 

As showed in Table 1, different enriched BP and CC are related with the ion transport across the membrane previously highlighted in the MF enriched. These results suggested that, in the cerebellum, AD pathology influences ion transport activity.

We expanded our analysis replicating it in the other two brain areas provided in the dataset, that are prefrontal cortex and visual cortex, to understand if the results obtained are related exclusively to cerebellum or also to other brain areas. We also checked if the DEGs reported in Figure 1 for cerebellum were present also in the prefrontal cortex and the visual cortex. In the prefrontal cortex, none of the 21 genes of Figure 1 resulted as DEGs. On the contrary, in the visual cortex, two of them resulted as DEGs as reported in Table S1. ORA for MF of prefrontal cortex and visual cortex comparisons showed that the MF “active ion transmembrane transporter activity” was not overrepresented. Specifically, the prefrontal cortex showed no MF overrepresented and for the visual cortex, the MF “lamin binding”, “disordered domain specific binding”, and “transcription coregulator binding” were overrepresented. Results of DEGs and ORA of the prefrontal cortex and the visual cortex suggest that, in an AD condition, ion transport imbalance can be specifically related to the cerebellum. 

Considering that gender differences in the incidence of AD are known, with a prevalence in female, we chose to repeat ORA of each tissue separating males from females and comparing them separately. ORA of MF for the cerebellum shows that only for the comparison of the females results MF overrepresented and, among these, some are related to ions, and specifically “ligand-gated cation channel activity”, “ligand-gated ion channel activity”, “ligand-gated channel activity”, “voltage-gated potassium channel activity”, “voltage-gated cation channel activity”, “voltage-gated ion channel activity”, “voltage-gated channel activity”, and “cation channel activity”. The result of ORA for the visual cortex showed no MF overrepresented among females while for males, several MFs were enriched, but none of them were correlated with ionic activity. Results of ORA for MF separated for sex are reported in Table S2.

## 3. Discussion

Utilizing whole-genome expression data is a valuable tool for gaining a deeper understanding of novel pathogenic pathways and predicting new diagnostic and therapeutic approaches in various clinical contexts, including neurodegenerative diseases [31,32]. Further elucidating the genetic underpinnings of AD could be crucial in establishing a foundation for the development of innovative diagnostic and therapeutic strategies [33].

In this study, we conducted a transcriptomic analysis of the cerebellum in both non-AD and AD patients, aiming to explore the role of this relatively understudied brain region in AD. We have analysed the DEGs in the cerebellum of 129 AD patients and 101 non-AD subjects. Interestingly, only one MF was significantly overrepresented, that is “active ion transmembrane transporter activity”. This result is of particular relevance considering that it is known that the regulation of ionic homeostasis is of pivotal importance for different neuronal functions. In particular, ion gradients are essential for intra- and inter-cellular communications within neuronal networks [30]. It is worth mentioning that Ca^2+^ signalling is implicated in the release of neurotransmitters as well as in the synaptic plasticity processes [30]. Instead, Na^+^ entry in neurons is fundamental for the trigger and the propagation of action potentials [30]. The efflux of potassium (K^+^) ions is involved in the repolarization of membrane potential after depolarization [30]. Of note, ionic homeostasis is altered during the cascade of events triggered by progressive Aβ overproduction and accumulation, leading to Ca^2+^ dyshomeostasis, K^+^ and Na^+^ channels deregulation, and inducing membrane depolarization [30]. In particular, the alteration of Na^+^ and Ca^2+^ homeostasis is involved in synaptic dysfunction and neuronal loss in AD [30].

Moreover, it should be noted that the remodeling of neuronal ionic homeostasis by altered ion channels and transporters is a pivotal characteristic of AD pathogenesis [34]. 

Then, we focused on the 21 DEGs involved in the significant MF, looking at the overrepresented BP in which they are involved. The majority of them are linked to the response and transport of metal and inorganic ions. Metal ion homeostasis is essential to maintaining brain physiology. Alterations in metal ion balance in the brain are related to Aβ deposition and tau hyperphosphorylation/accumulation, indicating a major role of metal ions in AD pathogenesis [35]. Interestingly, two of the BP overrepresented are linked to manganese (Mn) ions. Overexposure to Mn may induce neurotoxicity and may contribute to the development of AD, AD-like symptoms, or parkinsonism [36]. Interestingly, one of the 21 DEGs is the gene *SLC11A1*, that functions as a divalent transition metal (iron and Mn) transporter. Iron dyshomeostasis can also influence AD pathogenesis. In particular, increased iron was found at the highest levels both in the cortex and cerebellum from the pre-clinical AD/mild cognitive impairment cases [37], indicating that the cerebellum is involved in iron dyshomeostasis in AD.

Looking at the CC, we found that both axon, neuron projections and cell body are overrepresented. Moreover, also synapse related CC are overrepresented. Indeed, as already noted, ions play a major role in synaptic transmission and neurotransmitter release.

The 21 DEGS belonged to different protein families: ATP-binding cassette (ABC) transporters, P- and Vacuolar (V)-type ATPases, and solute carrier (SLC) transporters. 

ABC transporters are ATP dependent pumps and transport different endogenous substrates, such as inorganic and metal ions, peptides, amino acids, sugars, and hydrophobic compounds and metabolites. In particular, the Subfamily C of the ABC family included transporters associated with multidrug resistance [38]. Some members of this family were already shown to play a role in Aβ clearance [39]. 

P-type ATPases are essential ion-transporting pumps while V-type ATPase acidify different intracellular organelles and pump protons across the plasma membranes. *ATP1A3* was previously identified as one potential marker for AD diagnosis compared with vascular dementia [40]. It encodes for Na^+^/K^+^-ATPase α3 subunit (NAKα3). Na^+^/K^+^-ATPase α isoform plays a critical role in the regulation of learning and memory and the α3-mRNA was found significantly decreased in AD [41]. Aβ assemblies can target neuron-specific NAKα3 damaging its activity leading to mitochondrial Ca^2+^ dyshomeostasis, tau abnormalities, and neurodegeneration [42]. Also Tau fibrils cause a reduction in the amount of NAKα3. It was speculated that NAKα3 plays a role in Tau fibrils endocytosis and possibly in their subsequent amplification within neuron cytosol [43]. Contrary to previous studies, our analysis evidenced the upregulation of *ATP1A3* in the cerebellum of AD patients. 

Copper homeostasis is essential for proper brain functions and studies suggest an important role for copper in AD [44]. Copper can regulate *APP* expression and APP has a role in copper homeostasis. Fibroblasts overexpressing the Menkes protein, encoded by *ATP7A,* a major mammalian copper efflux protein, have severely depleted intracellular copper, while its deletion increases intracellular copper. Copper depletion reduced APP protein levels and down-regulated *APP* gene expression [45]. *ATP7A*, is highly expressed in activated microglial cells around amyloid plaques in a mouse AD model [46]. On the contrary, our analysis revealed its downregulation in the cerebellum of AD patients, which is in line with the previous study, and may led to an increase in copper levels. It is interestingly to notice that *ATP7A* levels were reported to be greater in the cerebellum than in other brain regions, indicating an important role for *ATP7A* in cerebellar neuronal health [47]. Then, its downregulation may be correlated to the pathological process of AD.

Mutations in the *ATP13A2* gene were associated to the Kufor–Rakeb syndrome (KRS), an early-onset autosomal recessive form of Parkinson’s disease with dementia [48].

The SLC group of membrane transport proteins includes over 400 members mostly located in the cell membrane. In line with our results, a study reported that the expression of *SLC3A2* was elevated in the isolated brain microvessels of a mouse model of familial AD [49] and in the brain cortical tissue of male TgF344-AD rats [50] compared to respective controls. We also found the downregulation of *SLC4A10*. *SLC4A10* is a major contributor to CSF secretion, and it was hypnotized that it may play a role in AD [51,52]. Indeed, the compromised function of the choroid plexus and cerebrospinal fluid production and turnover may play a role in AD due to the reduced clearance of Aβ. Then, reduced cerebrospinal fluid turnover may represent a risk factor for AD.

Interestingly, a family-based genome wide association study has shown a significant association of a rare single nucleotide polymorphism on the *SLC8A3* (alias NCX3) gene with the age at onset of AD [30,53]. It has been found that the NCX3 mRNA and protein levels were reduced in the hippocampus in an AD mouse model and that *NCX3^+/−^* mice showed cognitive deficits [54,55]. Interestingly, the aggregation of Aβ_1–42_ plaques in neurons could cause the NCX3 downregulation [56]. Furthermore, a study on postmortem frontal cortex of AD patients suggested that Aβ mediates calpain cleavage of NCX3 in the brain of AD patients, indicating that the decreased activity of NCX3 might contribute to the rise in intraneuronal Ca^2+^ concentrations, associated with synaptic and neuronal dysfunction in AD [57]. Interestingly, *Withania somnifera* (L.) Dunal treatment exerted neuroprotective effects in an AD mouse model through the rectification of NCX3 expression, protecting against the Ca^2+^ dyshomeostasis induced neuronal cell death [56]. Indeed, the upregulation of NCX3 is auspicable in AD and the pharmacological stimulation of the activity of the NCX isoforms, such as NCX3, might be a promising valuable strategy to ameliorate the course of several neurological diseases, including AD [58].

However, a study that analysed the synaptosomal expression of NCX3 in the parietal cortex of late-stage AD patients revealed that NCX3 co-localized with Aβ in synaptic terminals and was up-regulated in pathological terminals that contained Aβ [59]. Higher levels of NCX3 in Aβ-positive terminals could follow oligomeric Aβ–induced Ca^2+^ imbalance and could suggest that NCX3 could be involved in Ca^2+^ homeostasis in surviving synapses affected by the intraterminal toxicity of Aβ oligomers [59]. In addition, in agreement with these data, a study in an in vitro model of AD has suggested that the NCX3-mediated replenishment of the endoplasmic reticulum Ca^2+^ stores is a pivotal mechanism acting in neuronal homeostasis and supporting neuronal survival under pathological conditions, such as those triggered by Aβ_1–42_ oligomers [34]. Moreover, it has also been demonstrated that the Aβ_1–42_, via Ca^2+-^dependent calpain activation, generates a hyperfunctional form of NCX3 that contributes to increase the Ca^2+^ content in the endoplasmic reticulum, thus delaying caspase-12 activation and neuronal death and potentially representing a first defense mechanism against Aβ_1–42_-insult [60]. Hence, it seems that the expression and the role of NCX3 could be different between different brain regions. Specifically, in the cerebellum, our analysis revealed *SLC8A3* upregulation.

The other SLC members that we found deregulated were not associated to AD to our knowledge, but are involved in the transport of metal ions, organic cations and anions, Aspartate/Glutamate, Sodium-Phosphate, and amino acids.

The analysis revealed also a downregulation of *KCNJ8*. It was reported that serum levels KCNJ8 correlate with cortical amyloid deposition and may be useful for identifying elderly individuals at AD risk [61].

Then, our results suggest that some of the DEGs already reported as altered in AD showed an abnormal expression also in the cerebellum. However, while in some cases such as for *SLC3A2*, the expression assumed the same regulation, other genes assumed an opposite expression. In this regard, the upregulation of *ATP3A1* and *SLC8A3* genes in the cerebellum of AD patients could be a mechanism involved in a survival strategy against the pathological processes involved in AD. Indeed, it should be noted that the cerebellum can be considered a survivor of the preclinical stage of AD, thus suggesting that it might be resistant to specific neurodegenerative mechanisms and that it could have some protective mechanisms against AD pathology and could be an area of interest for neuroprotective pathways [26,62]. Indeed, it is known that, while at the beginning, amyloid plaques are present in the cerebral cortex, when the disease evolves, they propagate to the subcortical regions and, in the last stages, to the cerebellum [24]. Alterations such as the upregulation of *SLC11A1* and the downregulation of *ATP7A* can be involved in the increase in metal ions and can be associated to the AD pathology. 

Interestingly, the MF active ion transmembrane transporter activity was not enriched either in the visual cortex or in the prefrontal cortex, indicating that it represents a peculiarity of the cerebellum. This result suggests that the cerebellum may be particularly sensitive to changes in ion homeostasis, in line with the already indicated reports that highlight the importance of *ATP7A* in cerebellar neuronal health [47] and the increased cerebellar iron in pre-clinical AD/mild cognitive impairment cases [43]. 

We also performed analysis by separating male and female subjects in the AD and non-AD groups. Indeed, differences in the AD incidence in the two genders are reported. In particular, the prevalence of AD is higher in females compared to males [63]. In line, we observed that only in the female group analysis and only for the cerebellum, MF regarding ions channels were overrepresented. This result highlights the importance of ion homeostasis in the cerebellum in AD pathology.

In this study, we found new DEGs that may be involved in the cerebellum response to AD pathology. However, a limitation is that the existing interindividual variability may influence the data. Moreover, the results observed in this study should be also evaluated in vitro, studying the effects observed silencing the expression of each DEGs with transfection. Then, the results should also be confirmed in genetic AD in vivo models in order to deepen the knowledge on the role of these DEGs in the cerebellum in AD pathology. Indeed, it would be important to highlight whether these DEGs are correlated to the progression of the disease or if they are involved in the mechanism of resistance of the cerebellum in AD pathology.

## 4. Materials and Methods

### 4.1. Microarray Dataset Selection

The Gene Expression Omnibus (GEO) database is an international public repository established by the National Center for Biotechnology Information (NCBI) at the National Library of Medicine (NLM) that archives and makes freely available for download high-throughput microarray- and sequence-based functional genomic datasets submitted by the scientific community [64,65,66]. The GEO database, accessed on 8 February 2023, is freely accessible at http://www.ncbi.nlm.nih.gov/geo/ [65]. The database not only provides access to huge amounts of data, but also provides different web-based tools and strategies that assist users to query and analyze the data [65,66]. The availability of the high-throughput data in the GEO database is paving the way to novel perspectives and possibilities in scientific research [66]. Indeed, the possibility of reusing and reanalyzing large amounts of data thanks to the use of GEO database in order to validate new hypotheses and respond to different questions t from those posed in the initial studies represents a fundamental turning point for the scientific community [66].

In particular, it is worth pointing out that the genome-wide RNA or DNA microarray analysis has revolutionized biomedical research [67]. Microarrays have been extensively used to characterize disease-associated gene regulation but also gene expression patterns in different disease subtypes, as well as gene biomarkers of different diseases, including neurological disorders [67]. Different from the traditional biological assays, the use of microarrays allows the contemporaneous measure of tens of thousands of messenger RNA (mRNA) transcripts for gene expression as well as of genomic DNA fragments for copy number variation analysis [67]. 

All the data used for this study were retrieved from the GEO database (https://www.ncbi.nlm.nih.gov/geo/, accessed on 8 February 2023) searching for the keywords “Alzheimer” and “cerebellum”. We included, in the research, the transcriptomic data obtained for humans using high throughput sequencing or expression profile by array. Considering the wealth of information and the number of samples included, we selected the experiment with GEO accession GSE44768. This dataset is a sub series of a super series that include, for the same cohort, the data related to two additional tissues: dorsolateral prefrontal cortex and visual cortex; to enrich our analysis, we downloaded the super series GSE44772 [10]. Detailed description of the experimental design and procedures can be obtained from the publication by Zhang et al. [10].

### 4.2. Cohort Information

The dataset collects information about age, gender, condition, and post mortem interval (PMI) reported in hours about 230 samples of which 101 non-AD and 129 AD. This study included only individuals of Caucasian ancestry. In addition to ethnicity information, the dataset reports data about sex, age, and post-mortem interval (PMI) related to each sample. Distributions of the characteristics above mentioned are reported in Table 2. 

The preservation methods used to maintain the brain samples described above was liquid nitrogen vapor for 195 samples (84.8%), whereas for 35 samples (15.2%), dry ice was used. From each of the 230 brains, tissues from 3 different areas were collected: the cerebellum, the prefrontal cortex, and the visual cortex. Before the analysis step of the different areas for each of 230 samples, data about pH and RNA integrity number (RIN) were reported as described in Table 3. All the data reported above were used in the bioinformatic analysis as covariates to adjust, with more accuracy, the expression signals and include as many variables as possible. Comparisons were conducted among the same tissue of AD and non-AD group. Data related to pH and RIN of each area in the AD and non-AD conditions are reported in Table 3.

### 4.3. Biological Sample Treatment

For each tissue of the different samples, the total RNA was extracted using protocol Qiagen RNeasy spin columns with DNAse treatment. For the hybridization, the microarrays were incubated at 40 °C for 48 h in a rotating carousel, then they were washed to remove non-specific hybridized sample. RNA preparation and array hybridizations involved the utilization of custom microarrays produced by Agilent Technologies. These arrays were composed of 4720 control probes and 39,579 probes designed to target transcripts, encompassing 25,242 established genes and 14,337 predicted genes. For each of the brain tissue samples, 1 μg of total RNA was subjected to reverse transcription. Subsequently, the resulting cDNA was labeled using either Cy3 or Cy5 fluorochrome. The purified complementary RNA labeled with Cy3 or Cy5 was then subjected to hybridization on the microarrays, with fluorescence reversal, within a hybridization chamber. Following a 24 h hybridization period, the arrays were washed and subjected to scanning utilizing a laser confocal scanner.

### 4.4. Bioinformatic Analysis

Initially, the dataset related to a cerebellar area was obtained using the matrix composed of the list of the probes in the rows and the list of the samples on the columns. All the analyses implemented to observe the DEGs were performed using R v. 4.2.2 (R Core Team) with the package limma v. 3.54.1 [68] of Bioconductor v. 3.16 [69]. For transcriptomic, data initially performed a background correction using the function “backgroundCorrect” to calibrate the data for the ambient intensity that encompasses each feature. The second step was normalization performed using the function “normalizeQuantiles” and choosing the normalization per quantile. For normalized, data were removed from the different batch effects determined by pH, RIN, conservation methods, and PMI of our samples through the function “removeBatchEffect” and, once finished, we made a contrast matrix. The data concerning age and sex of the samples from the two cohorts were treated as covariates. The aim was to adjust the expressions based on the variations in this data, thereby highlighting more accurately the DEGs relevant to our specific condition of interest: AD or non-AD. The function “makeContrasts” was performed to obtain the fold changes of the DEGs and q-value thus associated. The q-value threshold used to deem the DEGs statistically significant was 0.05. The list of DEGs was used as an input to perform the ORA for the gene ontology through the package “clusterProfiler” v. 4.6.2 [70]. This analysis highlights the gene ontologies that were statistically significantly altered. The list of overrepresented ontologies gives us information on where the transcriptomic alterations were concentrated and which processes were most affected by AD. All the analyses reported above were performed initially on the cerebellum and later on the prefrontal cortex and the visual cortex.

## 5. Conclusions

Our study contributes to deepen the knowledge on the cerebellum in AD, highlighting that ion transmembrane transporter activity was altered in AD cerebellum compared to controls. Some of the DEGs were already reported as altered in AD, with the same or opposite regulation. Some DEGs may be involved in ion dyshomeostasis contributing to AD pathology. On the contrary, some of them such as *SLC8A3* and *ATP3A1* may be associated with a protective phenotype, that may explain the initial resistance to neurodegenerative mechanisms in AD. Additional preclinical studies in AD cellular and animal models and clinical studies are needed in order to better characterize the role of the cerebellum in AD and to contribute to paving the way to novel possible perspectives in the diagnostic and therapeutic settings.

## Figures and Tables

**Table 1:** Overrepresented BP and CC that includes analysed DEGs.

GO	ID	Description	DEGs
BP	GO:0021860	pyramidal neuron development	*ATP7A*, *SLC4A10*
	GO:0021859	pyramidal neuron differentiation	*ATP7A*, *SLC4A10*
	GO:0071287	cellular response to manganese ion	*ATP13A2*
	GO:0010042	response to manganese ion	*ATP13A2*, *ATP7A*
	GO:1990573	potassium ion import across plasma membra	*ATP12A*, *ATP1A3*, *KCNJ8*, *SLC12A4*
	GO:0098659	inorganic cation import across plasma membrane	*ATP12A*, *ATP1A3*, *KCNJ8*, *SLC12A4*, *SLC34A1*, *SLC8A3*
	GO:0099587	inorganic ion import across plasma membrane	*ATP12A*, *ATP1A3*, *KCNJ8*, *SLC12A4*, *SLC34A1*, *SLC8A3*
	GO:0071248	cellular response to metal ion	*ATP13A2*, *ATP7A*, *SLC34A1*
	GO:0071241	cellular response to inorganic substance	*ATP13A2*, *ATP7A*, *SLC34A1*
	GO:0010038	response to metal ion	*ABCC6*, *ATP13A2*, *ATP7A*, *SLC25A13*, *SLC34A1*
CC	GO:0090533	cation-transporting ATPase complex	*ATP12A*, *ATP1A3*, *KCNJ8*
	GO:0098533	ATPase dependent transmembrane transport complex	*ATP12A*, *ATP1A3*, *KCNJ8*
	GO:0043679	axon terminus	*ATP1A3*, *SLC4A10*, *SLC8A3*
	GO:0044306	neuron projection terminus	*ATP1A3*, *SLC4A10*, *SLC8A3*
	GO:0043204	perikaryon	*ATP7A*, *SLC4A10*, *SLC8A3*
	GO:0016323	basolateral plasma membrane	*ABCC3*, *ABCC6*, *ATP12A*, *ATP7A*, *SLC22A8*, *SLC22A9*, *SLC3A2*, *SLC4A1*, *SLC4A10*
	GO:0098562	cytoplasmic side of membrane	*SLC4A1*
	GO:0009925	basal plasma membrane	*ABCC3*, *ABCC6*, *ATP12A*, *ATP7A*, *SLC22A8*, *SLC22A9*, *SLC3A2*, *SLC4A1*, *SLC4A10*
	GO:0045178	basal part of cell	*ABCC3*, *ABCC6*, *ATP12A*, *ATP7A*, *SLC22A8*, *SLC22A9*, *SLC3A2*, *SLC4A1*, *SLC4A10*
	GO:0150034	distal axon	*ATP1A3*, *SLC4A10*, *SLC8A3*
	GO:0043025	neuronal cell body	*ATP13A2*, *ATP1A3*, *ATP7A*, *SLC3A2*, *SLC4A10*, *SLC8A3*
	GO:0098984	neuron to neuron synapse	*ATP1A3*, *ATP7A*, *SLC8A3*
	GO:0014069	postsynaptic density	*ATP7A*, *SLC8A3*
	GO:0099572	postsynaptic specialization	*ATP7A*, *SLC8A3*
	GO:0045177	apical part of cell	*ATP12A*, *ATP6V0D2*, *ATP7A*, *SLC22A18*, *SLC22A8*, *SLC34A1*, *SLC3A2*, *SLC4A10*

The first columns describe whether the GO is referred to a BP or CC followed by the respective ID in the second column. In the third column, the descriptions of the ontologies are presented followed by the list of the DEGs included in those reported in Table 1.

**Table 2:** Sex, age, and PMI distributions for each condition.

Condition	Sex		Age	PMI
	F	M		
AD	67 (51.9%)	62 (48.1%)	80.1 ± 9.3	13.7 ± 7.6
non-AD	19 (18.8%)	82 (81.2%)	62.1 ± 10.8	22.4 ± 5.8

In the first column, the studied conditions are reported and for each of these mean ± SD of sex, age, and PMI are shown. Age and PMI data, reported in the third and fourth column, are SD rounded off to 1 decimal.

**Table 3:** pH and RIN related to the different brain areas for each condition.

Brain Area	Condition	pH	RIN
Cerebellum	AD	6.4 ± 0.3	6.7 ± 0.5
	non-AD	6.6 ± 0.2	6.7 ± 0.4
Prefrontal cortex	AD	6.3 ± 0.3	7.1 ± 0.6
	non-AD	6.6 ± 0.3	7.3 ± 0.5
Visual cortex	AD	6.3 ± 0.3	6.7 ± 0.6
	non-AD	6.5 ± 0.3	7.1 ± 0.5

The first columns report the three different brain areas and each of these is divided for each condition reported in the second column. The Third and fourth columns show pH and RIN with mean ± SD rounded off to 1 decimal.

## Data Availability

The data presented in this study are openly available in the Gene Expression Omnibus (GEO) database (https://www.ncbi.nlm.nih.gov/gds; accessed on 8 February 2023), under the accession number GSE44772.

## Supplementary Materials

The following supporting information can be downloaded at https://www.mdpi.com/article/10.3390/ijms241813924/s1.
