# Supplementary material for: In Silico Analysis Reveals the Modulation of Ion Transmembrane Transporters in the Cerebellum of Alzheimer’s Disease Patients
Source: Int J Mol Sci. 2023 Sep 10;24(18):13924. doi: 10.3390/ijms241813924 (PMC10530854; doi:10.3390/ijms241813924)
Supplement: Supplementary file 1 [file ijms-24-13924-s001.zip › Table S1.pdf]

Table S1. Fold changes and q value in the different brain areas of the 21 genes belonging to the MF "active ion transmembrane transporter activity", resulted as DEGs in cerebellum.

Gene	Cerebellum		Visual cortex		Prefrontal cortex	
	Fold Change	q value	Fold Change	q value	Fold Change	q value
ABCC3	0.013991175	0.018415761	-	> 0.05	-	> 0.05
ABCC6	0.012650605	0.04160476	-	> 0.05	-	> 0.05
ATP12A	-0.006174467	0.042804469	-	> 0.05	-	> 0.05
ATP13A2	0.251463979	0.035049666	-	> 0.05	-	> 0.05
ATP1A3	0.09634255	0.041888111	-	> 0.05	-	> 0.05
ATP6V0D2	-0.006374808	0.008250677	-	> 0.05	-	> 0.05
ATP7A	-0.015102459	0.036627874	-	> 0.05	-	> 0.05
KCNJ8	-0.012363192	0.047113632	-	> 0.05	-	> 0.05
SLC11A1	0.021200058	0.012683541	-	> 0.05	-	> 0.05
SLC12A4	2.499045432	0.018632738	0.0443270969614519	0.00827453181921639	-	> 0.05
SLC22A18	0.04532508	0.009564829	-	> 0.05	-	> 0.05
SLC22A8	0.014382656	0.009790237	-	> 0.05	-	> 0.05
SLC22A9	0.009494155	0.030875899	-	> 0.05	-	> 0.05
SLC25A13	-0.017539692	0.025486565	-	> 0.05	-	> 0.05
SLC34A1	0.013895368	0.018821196	-	> 0.05	-	> 0.05
SLC36A3	-0.00704993	0.007590429	-	> 0.05	-	> 0.05
SLC3A2	0.100642774	0.040843147	0.141893882928591	0.0109535278650002	-	> 0.05
SLC4A1	0.105817307	0.008598399	-	> 0.05	-	> 0.05
SLC4A10	-0.005783852	0.004750603	-	> 0.05	-	> 0.05
SLC8A3	0.014647883	0.031575018	-	> 0.05	-	> 0.05
SLCO1A2	0.03306782	0.03695757	-	> 0.05	-	> 0.05
